
JOURNAL INFORMATION
==============================
NLM Title Abbreviation: Behav Neurol
Journal ID: Behav Neurol
NLM Journal ID: 8914585
Journal ID: BN

Behavioural Neurology

ISSN: 0953-4180
EISSN: 1875-8584
Publisher: Wiley

ARTICLE INFORMATION
==============================
PMCID: PMC5215754
PMID: 23242354
DOI: 10.3233/BEN-129015
Article ID: 125401
Article version: 1
Subjects: Editorial

Towards a Deeper Comprehension of Relationships among Cognitive, Behavioral and Psychiatric Symptoms in Parkinson’s Disease

Trojano Luigi *
Santangelo Gabriella
Conson Massimiliano
Grossi Dario
Neuropsychology Lab, Department of Psychology, Second University of Naples, Caserta Italy
*Luigi Trojano: luigi.trojano@unina2.it
Print publication date: 2013
Volume: 27
Issue: 4
First page: 463
Last page: 467
Received 2012 Nov 23; Accepted 2012 Nov 23
Copyright: Copyright © 2013 Hindawi Publishing Corporation and the authors.
Copyright year: 2013
License: This is an open access article distributed under the Creative Commons Attribution License, which permits unrestricted use, distribution, and reproduction in any medium, provided the original work is properly cited.
License URL: https://creativecommons.org/licenses/by/3.0/

==============================
Behavioural Neurology 27 (2013) 463–467
DOI 10.3233/BEN-129015
IOS Press

Editorial

### Towards a deeper comprehension of relationships among cognitive, behavioral and psychiatric symptoms in Parkinson's disease

Luigi Trojano*, Gabriella Santangelo, Massimiliano Conson and Dario Grossi
*Neuropsychology Lab, Department of Psychology, Second University of Naples, Caserta, Italy*

Parkinson's Disease (PD) is nowadays considered as a complex clinical condition in which motor symptoms coexist with several non-motor symptoms. A growing body of literature has addressed non-motor symptoms in PD [1], that are progressively being recognized as a potential source of distress for patients and their families, even greater than that imposed by the well-known motor impairments [2–4].

The aim of this special issue was to gather together studies providing new findings about a specific subset of non-motor symptoms in PD: cognitive, behavioural and psychological disturbances, that are likely those with the highest impact on patient's daily life [5,6]. Within this framework we collected papers addressing neuropsychological defects (particularly frontal dysfunctions and dysexecutive syndromes) and neuropsychiatric disorders (such as hallucinations, depression and apathy, compulsive behavior), and highlighting the possible relationships of these symptoms with pharmacological treatment, and, above all, with each other.

In the present issue a review by Friedman et al. [7] addressed occurrence of psychosis and hallucinations in PD. Psychotic symptoms are common in drug treated PD patients: visual hallucinations (VH) have been reported to occur in about 30%, whereas delusions, typically paranoid in nature, occur in about 5%. These problems, particularly the delusions, are among the most important precipitants for nursing home placement, and are related with a poor prognosis (in terms of development of dementia and of increased mortality). There is considerable debate about the genesis of psychotic symptoms and their treatment options, since reduction of medication for PD motor symptoms and use of specific drug treatment for psychosis can both worsen motor function. At the moment there is level A evidence to support use of clozapine in PD patients with psychosis, with or without dementia, whereas quetiapine has been recommended for "consideration," since double blind placebo controlled trials have demonstrated safety but not efficacy.

The hypotheses about the genesis of VH in PD and Lewy body dementias have been reviewed by Onofrj et al. [8]. Classically, occurrence of VH in PD has been ascribed to visual disorders, cognitive deficits, Rapid Eye Movement (REM) Sleep Intrusions, dysfunctions of top down or bottom up visual pathways, or neurotransmitter imbalance [9–14]. As Onofrj et al. [8] underlined, more recent hypotheses introduce, among the possible mechanisms of VH, the role of attention networks and of the Default Mode Network (DMN), a network that is inhibited during attentional tasks and becomes active during rest and self referential imagery. Persistent DMN activity during active tasks with dysfunctional imbalance of dorsal and ventral attentional networks might represent a new hypothesis to explain the genesis of VH in PD.

*Corresponding author: Luigi Trojano, Neuropsychology Lab, Department of Psychology, Second University of Naples, Viale Ellittico 31, 81100 Caserta, Italy. E-mail: luigi.trojano@unina2.it.

ISSN 0953-4180/13/$27.50 © 2013 – IOS Press and the authors. All rights reserved

A brief report enclosed in the present issue by Cristinzio et al. [15] specifically tested the efficiency of three attentional systems (spatial orienting, phasic alerting and executive control) in patients with Parkinson's disease (PD), by means of a task employing acoustic tones to modulate phasic alertness. PD patients were generally slower than age-matched controls, but they showed a similar pattern of effects and interactions. In particular, as in normal subjects, PD patients' responses showed a normal congruency effect (thought to reflect executive control), and were faster for valid visual cues than with invalid or no cues (thus demonstrating a normal orienting effect), and when acoustic tones preceded the target (with a higher-than-normal alerting effect). Therefore, Cristinzio et al. [15] did not detect any selective impairment of the attentional networks in PD patients without dementia and without VH, but observed a general slowing of responses in PD, that was at least partially ascribed to bradykinesia. Further specifically designed studies are necessary to demonstrate the behavioral correlates of putative attentional defects in the genesis of VH.

A possible role of cognitive alterations (e.g. attentional or executive impairments) has been hypothesized also for the genesis of other psychological disorders in PD. In the present issue a review by Santangelo et al. [16] specifically addressed apathy, i.e. lack of motivation, that is associated with decreased daily function, and increased stress for families [5,17]. Apathy has been traditionally considered as a symptom of psychiatric disorders, such as major depression and schizophrenia, but more recently it has been recognized as a specific neuropsychiatric syndrome associated with neurodegenerative diseases such as PD. The overlap between apathy and depression can cause substantial difficulties in identifying and distinguishing the two syndromes [18,19]. Although most studies found an association between apathy and depression in PD patients, and between apathy and anhedonia, "pure" apathy can be recognized in PD when specific diagnostic tools are employed [20,21]. As underlined by Santangelo et al. [16], apathy and depression can dissociate in PD, and are characterized by different features: depression includes sadness and negative thoughts about the self, while diagnosis of apathy rests on lack of initiation and lack of effort, but not on negative self or event appraisal. Although neuroimaging studies do not provide a unique anatomic pattern, several data suggest that the ventromedial prefrontal cortex and the basal ganglia connected through frontal-subcortical circuits, are particularly involved in the genesis of apathy. This hypothesis about the neural bases of apathy received support from neuropsychological studies highlighting the presence of frontal/executive disorders in PD patients with "pure apathy" (i.e., non-demented, non-depressed patients with high scores on specific questionnaires for apathy) [22,23]. An original research paper by Grossi et al. in the present issue [24] verified that this specific relationship holds true in both demented PD patients and patients with Alzheimer's disease. Actually, Grossi et al. [24] explored memory, visuospatial and executive functions in patients affected by the two neurodegenerative diseases; the authors also used specific behavioral rating scales and an inventory developed to measure severity of apathy on clinical basis, the newly proposed Clinical Judgment for Apathy Syndrome. The results showed that apathetic patients with both forms of dementia showed a common neuropsychological and behavioral picture, characterized by impairment on frontal tasks, thus strongly supporting the existence of an 'apathetic syndrome', characterized by specific cognitive and psychological symptoms.

A further research paper enclosed in the present issue by Costa et al. [25] explored the impact of dysexecutive defects on the ability to understand the mental states of others, as assessed by Theory of Mind (ToM) tasks in non-demented PD patients. Dysexecutive PD patients performed less accurately on a faux pas recognition test administered in written modality with respect to PD patients without dysexecutive impairment; this latter group of PD patients performed as accurately as the normal control group. According to the authors, these results would demonstrate that PD is not necessarily associated with ToM impairments, but that only PD patients with executive defects show an impaired ability to understand others' mental state, in agreement with recent findings showing a significant association between impaired cognitive aspect of ToM abilities and frontal dysfunction in PD patients without dementia [26].

Besides the relationships between apathy and dysexecutive impairment, another possible correlate of apathy has been explored by Bogdanova and Cronin-Golomb in the present issue [27]. In fact the authors aimed at exploring the relation between apathy and alexithymia, an impairment of affective and cognitive emotional processing, mainly characterized by difficulties in identifying and describing own feelings, and increasingly recognized as a non-motor symptom in PD [28,29]. Bogdanova and Cronin-Golomb administered rating scales assessing neuropsychiatric status, including alexithymia, apathy, and depression, and a series of neuropsychological tests to non-demented PD patients and to a group of matched normal control adults. As expected, PD patients showed more alexithymia than normal controls, and there was a significant association between alexithymia and disease stage. Moreover, alexithymia was associated with performance on non-verbally mediated measures of executive and visuospatial function, but not on verbally mediated tasks, thus suggesting the possible association of alexithymia with non-verbal cognition, i.e. mainly with right hemisphere processes, in PD. Incidentally, Bogdanova and Cronin-Golomb did not find any significant correlation between cognition and ratings of apathy, apparently in contrast with the previous reviewed neuropsychological evidence. However, since alexithymia was correlated with severity of apathy in this sample, the relationships among alexithymia, apathy and dysexecutive functions remain to be fully elucidated.

A further kind of behavioral symptoms in PD, the impulse control disorders (ICDs), have been addressed by Bugalho and Oliveira-Maia in the present issue [30]. ICDs are characterized by an inappropriate drive to conduct repetitive behaviours that are usually socially inadequate or result in harmful consequences. In their review Bugalho and Oliveira-Maia discussed the association of ICDs with psychiatric co-morbidities such as obsessive-compulsive disorder and with dopamine related side effects, such as hallucinations and dyskinesias, the relationship with executive cognitive dysfunction [31,32], and the neural underpinnings of ICDs in PD. Taking into account evidence gathered from animal models, Bugalho and Oliveira-Maia proposed an explanation of the genesis of ICDs in PD specifically based on an alteration of corticostriatal control of goal directed and habitual actions.

Two interesting examples of ICDs in PD are provided by two case reports included in the present issue. Vitale et al. [33] described a PD patient who developed an unusual, severe, repetitive behavior characterized by an irrepressible need to drum and beat percussion instruments (compulsive drumming). This compulsive behavior was apparently related to introduction of pramipexole, and was not associated to a pattern of chronic inappropriate overuse of dopaminergic medication or other psychiatric symptoms. Therefore, compulsive drumming might be considered a distinct manifestation of punding, i.e. of aimless stereotyped behaviors performed for long periods of time at the expenses of other habitual daily activities [34] in PD. Solla et al. [35] reported, instead, on the dopamine dysregulation syndrome (DDS), an addiction-like behavioral disturbance developed by a small proportion of PD patients under chronic dopamine replacement therapy. This behavioral disorder is characterized by the increase of doses beyond those required for motor control, and its management remains difficult; thus, early recognition and careful monitoring of at-risk individuals are crucial. Solla et al. [35] described two PD patients with a previous unsatisfactory switching from an immediate release to an extended release pramipexole formulation who developed DDS. According to the authors an unsatisfactory switching to extended-release formulations of dopamine agonists in PD patients should be regarded as a potential index to identify individuals at-risk for DDS development.

To enrich the complex relationships among psychiatric, behavioral and cognitive disorders in PD, it is important to consider that PD patients may present with a personality profile characterized by low Novelty Seeking and high Harm Avoidance [36]. This profile can be identified as the disease emerges, which raises the question whether these traits correlate with more fundamental neuropsychological and emotional disturbances. In the present issue Koerts et al. [37] searched for neuropsychological and emotional correlates of Novelty Seeking, Harm Avoidance and of two other personality traits that are often considered in PD, i.e. Reward Dependence and Persistence. By means of a specific assessment tool, the Temperament and Character Inventory [38], Koerts et al. [37] observed a higher Harm Avoidance in PD patients than in healthy participants, whereas the two groups did not differ for Novelty Seeking, Reward Dependence and Persistence. In PD patients Harm Avoidance was predicted by symptoms of depression, Reward Dependence by cognitive flexibility, and Persistence by divergent thinking and inhibition. On these bases Koerts et al. [37] suggested that cognition and emotion are selectively related to personality traits in PD, thus implying that alterations in personality, cognition and emotion in PD would not be independent from each other.

Taken together, the papers enclosed in the present issue underline the current efforts to comprehend the nature of the relationships among cognitive, behavioral and psychiatric non-motor symptoms in PD. Further studies are necessary to verify whether the different symptoms share common pathogenetic mechanisms and whether they can be traced back to specific molecular alterations. We hope that the present issue will provide a valuable contribution towards this goal.

### References

[1] Weintraub D, Burn DJ. Parkinson's disease: The quintessential neuropsychiatric disorder. Mov Disord. 2011; 26(6): 1022-1031.

[2] Schrag A, Hovris A, Morley D, Quinn N, Jahanshahi M. Caregiver-burden in parkinson's disease is closely associated with psychiatric symptoms, falls, and disability. Parkinsonism Relat Disord. 2006; 12(1): 35-41.

[3] Aarsland D, Larsen JP, Karlsen K, et al. Mental symptoms in Parkinson's disease are important contributors to caregiver distress. Int J Geriatr Psychiatry. 1999; 14(10): 866-874.

[4] Aarsland D, Larsen JP, Lim NG, et al. Range of neuropsychiatric disturbances in patients with Parkinson's disease. J Neurol Neurosurg Psychiatry. 1999; 67(4): 492-496.

[5] Barone P, Antonini A, Colosimo C, Marconi R, Morgante L, Avarello TP, Bottacchi E, Cannas A, Ceravolo G, Ceravolo R, Cicarelli G, Gaglio RM, Giglia RM, Iemolo F, Manfredi M, Meco G, Nicoletti A, Pederzoli M, Petrone A, Pisani A, Pontieri FE, Quatrale R, Ramat S, Scala R, Volpe G, Zappulla S, Bentivoglio AR, Stocchi F, Trianni G, Dotto PD; PRIAMO study group. The PRIAMO study: A multicenter assessment of nonmotor symptoms and their impact on quality of life in Parkinson's disease. Mov Disord. 2009; 24(11): 1641-1649.

[6] Havlikova E, van Dijk JP, Nagyova I, Rosenberger J, Middel B, Dubayova T, Gdovinova Z, Groothoff JW. The impact of sleep and mood disorders on quality of life in Parkinson's disease patients. J Neurol. 2011; 258(12): 2222-2229.

[7] Friedman JH. Parkinson disease psychosis: Update. Behav Neurol. Present issue.

[8] Onofrj M, Taylor JP, Monaco D, Franciotti R, Anzellotti F, Bonanni L, Onofrj V, Thomas A. Visual Hallucinations in PD and Lewy body dementias: Old and new hypotheses. Behav Neurol. Present issue.

[9] Barnes J, Boubert L, Harris J, Lee A, David AS. Reality monitoring and visual hallucinations in Parkinson's disease. Neuropsychologia. 2003; 41(5): 565-574.

[10] Grossi D, Trojano L, Pellecchia MT, Amboni M, Fragassi NA, Barone P. Frontal dysfunction contributes to the genesis of hallucinations in non-demented Parkinsonian patients. Int J Geriatr Psychiatry. 2005; 20(7): 668-673.

[11] Santangelo G, Trojano L, Vitale C, Ianniciello M, Amboni M, Grossi D, et al. A neuropsychological longitudinal study in Parkinson's patients with and without hallucinations. Mov Disord. 2007; 22(16): 2418-2425.

[12] Diedrich NJ, Goetz CG, Raman R, Pappert EJ, Leurgans S, Piery V. Poor visual discrimination and visual hallucinations in Parkinson's Disease. Clin Neuropharmacol. 1998; 21(5): 289-95.

[13] Ramírez-Ruiz B, Martí MJ, Tolosa E, Falcón C, Bargalló N, Valldeoriola F, et al. Brain response to complex visual stimuli in Parkinson's patients with hallucinations: A functional magnetic resonance imaging study. Mov Disord. 2008; 23(16): 2335-2343.

[14] Manganelli F, Vitale C, Santangelo G, Pisciotta C, Iodice R, Cozzolino A, Dubbioso R, Picillo M, Barone P, Santoro L. Functional involvement of central cholinergic circuits and visual hallucinations in Parkinson's disease. Brain. 2009 Sep; 132(Pt 9): 2350-2355.

[15] Cristinzio C, Bononi M, Piacentini S, Albanese A, Bartolomeo P. Attentional networks in Parkinson's disease. Behav Neurol. Present issue.

[16] Santangelo G, Trojano L, Barone P, Errico D, Grossi D, Vitale C. Apathy in Parkinson's Disease: diagnosis, neuropsychological correlates, pathophysiology and treatment. Behav Neurol. Present issue.

[17] Benito-León J, Cubo E, Coronell C. ANIMO Study Group. Impact of apathy on health-related quality of life in recently diagnosed Parkinson's disease: the ANIMO study. Mov Disord. 2012; 27(2): 211-218.

[18] Schrag A, Barone P, Brown RG, Leentjens AF, McDonald WM, Starkstein S, Weintraub D, Poewe W, Rascol O, Sampaio C, Stebbins GT, Goetz CG. Depression rating scales in Parkinson's disease: critique and recommendations. Mov Disord. 2007; 22(8): 1077-1092.

[19] Santangelo G, Vitale C, Trojano L, Longo K, Cozzolino A, Grossi D, Barone P. Relationship between depression and cognitive dysfunctions in Parkinson's disease without dementia. J Neurol. 2009; 256(4): 632-638.

[20] Leentjens AF, Dujardin K, Marsh L, Martinez-Martin P, Richard IH, Starkstein SE, Weintraub D, Sampaio C, Poewe W, Rascol O, Stebbins GT, Goetz CG. Apathy and anhedonia rating scales in Parkinson's disease: Critique and recommendations. Mov Disord. 2008; 23(14): 2004-2014.

[21] Kirsch-Darrow L, Marsiske M, Okun MS, Bauer R, Bowers D. Apathy and depression: Separate factors in Parkinson's disease. J Int Neuropsychol Soc. 2011; 17(6): 1058-1066.

[22] Varanese S, Perfetti B, Ghilardi MF, Di Rocco A. Apathy, but not depression, reflects inefficient cognitive strategies in Parkinson's disease. PLoS One. 2011; 6(3): e17846.

[23] Dujardin K, Sockeel P, Delliaux M, Destée A, Defebvre L. Apathy may herald cognitive decline and dementia in Parkinson's disease. Mov Disord. 2009; 24(16): 2391-2397.

[24] Grossi D, Santangelo G, Barbarulo AM, Vitale C, Castaldo G, Proto MG, Siano P, Barone P, Trojano L. Apathy and related Executive Syndromes in Dementia associated with Parkinson's Disease and in Alzheimer's Disease. Behav Neurol. Present issue.

[25] Costa A, Peppe A, Martini M, Coletta K, Oliveri M, Caltagirone C, Carlesimo GA. Parkinsonian patients with deficits in the dysexecutive spectrum are impaired on theory of mind tasks. Behav Neurol. Present issue.

[26] Santangelo G, Vitale C, Trojano L, Errico D, Amboni M, Barbarulo AM, Grossi D, Barone P. Neuropsychological correlates of theory of mind in patients with early Parkinson's disease. Mov Disord. 2012; 27(1): 98-105.

[27] Bogdanova Y, Cronin-Golomb A. Alexithymia and apathy in Parkinson's Disease: Neurocognitive correlates. Behav Neurol. Present issue.

[28] Poletti M, De Rosa A, Bonuccelli U. Affective symptoms and cognitive functions in Parkinson's disease. J Neurol Sci. 2012; 317(1-2): 97-102.

[29] Bagby RM, Taylor GJ. Affect dysregulation and alexithymia. In: Taylor GJ, Bagby RM, Parker JDA, editors. Disorders of affect regulation Alexithymia in medical and psychiatric illness. Cambridge (UK): Cambridge University Press, 1997, pp. 26-45.

[30] Bugalho P, Oliveira-Maia AJ. Impulse control disorders in Parkinson's disease: Crossroads between Neurology, Psychiatry and Neuroscience. Behav Neurol. Present issue.

[31] Vitale C, Santangelo G, Trojano L, Verde F, Rocco M, Grossi D, Barone P. Comparative neuropsychological profile of pathological gambling, hypersexuality, and compulsive eating in Parkinson's disease. Mov Disord. 2011; 26(5): 830-836.

[32] Santangelo G, Vitale C, Trojano L, Verde F, Grossi D, Barone P. Cognitive dysfunctions and pathological gambling in patients with Parkinson's disease. Mov Disord. 2009; 24(6): 899-905.

[33] Vitale C, Trojano L, Barone P, Errico D, Agosti V, Sorrentino G, Grossi D, Santangelo G. Compulsive drumming induced by dopamine agonists treatment in Parkinson's disease: Another aspect of punding. Behav Neurol. Present issue.
[34] Spencer AH, Rickards H, Fasano A, Cavanna AE. The prevalence and clinical characteristics of punding in Parkinson's disease. Mov Disord. 2011; 26(4): 578-586.
[35] Solla P, Cannas A, Corona M, Marrosu MG, Marrosu F. Dopamine dysregulation syndrome in Parkinson's disease patients with unsatisfactory switching from immediate to extended release pramipexole: A further clue to incentive sensitization mechanisms? Behav Neurol. Present issue.
[36] Poletti M, Bonuccelli U. Personality traits in patients with Parkinson's disease: Assessment and clinical implications. J Neurol 2011; 17(6): 1158-1162.
[37] Koerts J, Tucha L, Leenders KL, Tucha O. Neuropsychological and emotional correlates of personality traits in Parkinson's disease. Behav Neurol. Present issue.
[38] Cloninger CR, Svrakic DM, Przybeck TR. A psychobiological model of temperament and character. Arch Gen Psychiatry. 1993; 50(12): 975-990.
